# Water Increases the Faradaic Selectivity of Li-Mediated Nitrogen Reduction

**DOI:** 10.1021/acsenergylett.2c02792

**Published:** 2023-01-31

**Authors:** Matthew Spry, Olivia Westhead, Romain Tort, Benjamin Moss, Yu Katayama, Maria-Magdalena Titirici, Ifan E. L. Stephens, Alexander Bagger

**Affiliations:** † Department of Materials, Imperial College London, Prince Consort Road, South Kensington, London, SW7 2AZ, U.K.; ‡ Department of Chemical Engineering, Imperial College London, Imperial College Rd, South Kensington, London, SW7 2AZ, U.K.; § SANKEN (Institute of Scientific and Industrial Research), Osaka University, Mihogaoka, Ibaraki, Osaka 567-0047, Japan

## Abstract

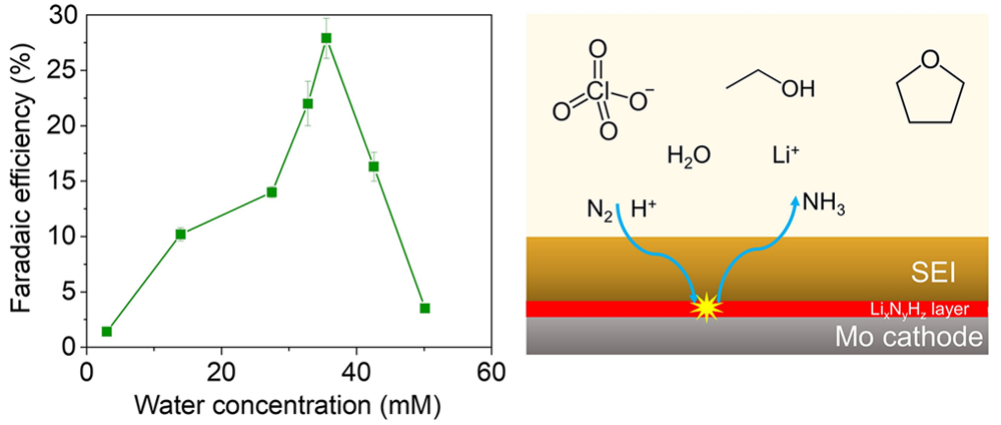

The lithium-mediated system catalyzes nitrogen to ammonia under ambient conditions. Herein we discover that trace amount of water as an electrolyte additive—in contrast to prior reports from the literature–can effect a dramatic improvement in the Faradaic selectivity of N_2_ reduction to NH_3_. We report that an optimal water concentration of 35.9 mM and LiClO_4_ salt concentration of 0.8 M allows a Faradaic efficiency up to 27.9 ± 2.5% at ambient pressure. We attribute the increase in Faradaic efficiency to the incorporation of Li_2_O in the solid electrolyte interphase, as suggested by our X-ray photoelectron spectroscopy measurements. Our results highlight the extreme sensitivity of lithium-mediated N_2_ reduction to small changes in the experimental conditions.

The lithium-mediated system of electrochemical nitrogen reduction has emerged as a promising alternative to the highly carbon-intensive steam methane reforming and Haber–Bosch process for manufacturing ammonia. It is to date the only rigorously verified method of electrochemical ammonia synthesis that has been reproduced by multiple laboratories.^1−7^ In this system, nitrogen is reduced to ammonia in an aprotic organic solvent containing a lithium salt and an organic proton source. Most reports attribute the ability of this system to synthesize ammonia to the ability of lithium and nitrogen to form lithium nitride under ambient conditions.^1,6,7^ The precise mechanism is not yet understood. Some reports propose that lithium nitride is directly protonated to ammonia, recycling the deposited lithium;^5,8,9^ others propose that the layer of Li, or a mixed Li_*x*_N_*y*_H_*z*_ layer, acts as an active surface for N_2_ reduction.^6,7^ In either case, nitrogen reduction must compete with hydrogen evolution and excessive lithium plating as parasitic side reactions; hence, there is a need to balance these competing reactions. Notably, the overpotentials for nitrogen reduction and hydrogen evolution are high due to the reaction operating at lithium-plating potential.^4,6,10,11^

We can understand lithium-mediated N_2_ reduction by drawing insight from lithium-ion batteries: in both systems, a solid-electrolyte interphase (SEI) forms as a result of decomposition of electrolyte components at the highly reducing potentials. The SEI is an electrically insulating passivation layer which restricts further electrolyte degradation.^12,13^ Furthermore, the SEI is lithium-ion conductive, and allows protons, nitrogen, and ammonia to pass through.^14^ Earlier studies have proposed that efficient nitrogen reduction can be achieved in the lithium-mediated system by controlling the transport of species to the electrode surface to suppress these side reactions.^6,15^ There is growing evidence that the key to controlling the rate of transport of nitrogen, lithium, and protons to the surface lies in modifying the solid-electrolyte interphase (SEI) layer that controls the mobility of these species^14,10^ However, the structure of the SEI and its function as a transport barrier is poorly understood in both this system and in batteries.^16,17^

Since the verification of the unoptimized system of Tsuneto et al. in 2019,^1,8^ which reported a maximum Faradaic efficiency of 7.8% at ambient pressure and 48% under 50 bar N_2_, several groups have reported significant improvements to the system in terms of selectivity and stability.^3−7,14,18−21^ The most obvious method of improving selectivity toward nitrogen reduction is to increase the N_2_ partial pressure: A number of additional strategies have been employed at elevated pressures, including potential cycling,^6^ utilizing a proton shuttle,^5^ and SEI tailoring,^14^ and selectivity of over 98% has been recently reported using a 2 M solution of lithium bis(trifluoromethylsulfonyl)imide (LiNTf_2_) in tetrahydrofuran (THF) and 0.1 M ethanol under 15 bar N_2_.^19^ At ambient pressure, the Faradaic efficiencies are lower. Currently, the highest reported Faradaic efficiency at ambient pressure is 35%, using a gas diffusion electrode (GDE) to circumvent N_2_ solubility challenges instead of increasing the pressure, in an electrolyte of 1 M LiBF_4_ in THF with 1% ethanol as a proton source.^21^

Despite considerable focus on the lithium-mediated system of N_2_ reduction in recent years, and the many factors that influence its performance, the effect of water has been somewhat overlooked. Tsuneto et al., in their seminal study on this system, report that water is ineffective as a proton source, instead recommending ethanol and taking measures to exclude water, measuring approximately 10 mM water in their electrolytes.^1^

As such, subsequent studies have excluded H_2_O as much as possible and study the reaction in the presence of around 2 mM water. The few studies which have examined the effect of water concur with the findings of Tsuneto et al. and also show that there is either no correlation between performance and water content or that it is detrimental to Faradaic selectivity (see Supporting Information).^1,3,4,22^ In our current study, we revisit these earlier studies, to systematically investigate how a controlled amount of trace water affects the performance of the system in terms of Faradaic efficiency for ammonia production at ambient nitrogen pressures in LiClO_4_ electrolytes, using ethanol as the proton source.

Water concentration was controlled by making up the electrolyte with varying ratios of dry and preprepared THF with a known water concentration. The water content of the electrolyte was measured using a Karl Fischer titration from aliquots taken immediately before and after each experiment (for details, see Supporting Information). Figure 1a shows the Faradaic efficiency trends for four salt concentrations with varying water concentration. As the water concentration increases over the course of each experiment (see Figure S2), the reported water content is the initial concentration measured in fresh electrolyte before each experiment. In each case, a maximum Faradaic efficiency is observed in the range of 25–50 mM. As salt concentration is increased, the maximum observed Faradaic efficiency occurred at higher water concentrations, from 14.6 ± 0.8% in 0.2 M LiClO_4_ electrolyte with 21.0 mM water, to 21.5 ± 1.8% in 1 M LiClO_4_ with 43.0 mM water. The maximum Faradaic efficiency across the experiments was 27.9 ± 2.5%, for 35.9 mM water with a salt concentration of 0.8 M. Due to the apparent sharpness of the peaks in Faradaic efficiency, and the difficulty in fine-tuning such a small water concentration when preparing the electrolytes, it is possible that even higher Faradaic efficiencies are possible with only slightly different salt, water, and ethanol concentrations to those tested here. Other Li-mediated experiments have been carried out using other anions, such as LiBF_4_, LiOTf, and LiNTf_2_ which appear to show improved performance over LiClO_4_.^3,4,19,21^ As these anions are fluorinated, they may generate solution phase HF upon reaction with water, promoting LiF formation in the SEI.^22,23^ Investigation of the effect of water concentration in these electrolytes is beyond the scope of this investigation, but is an interesting avenue for further study. Figure 1b shows a heat map of Faradaic efficiency against both water concentration and salt concentration, based on interpolations between measured results. Such a two-dimensional mapping of the system performance to controllable electrolyte parameters gives fundamental insights into the sensitivity of the system. While we observe a broad plateau of Faradaic efficiency with salt and water concentration around 15% Faradaic efficiency, the peak performance is limited to a very narrow range of optimal parameters. As shown in Figure 2, the maximum Faradaic efficiency we observed is, to the best of our knowledge, the highest Faradaic efficiency reported at ambient pressure so far without using a gas diffusion electrode.

**Figure 1:**
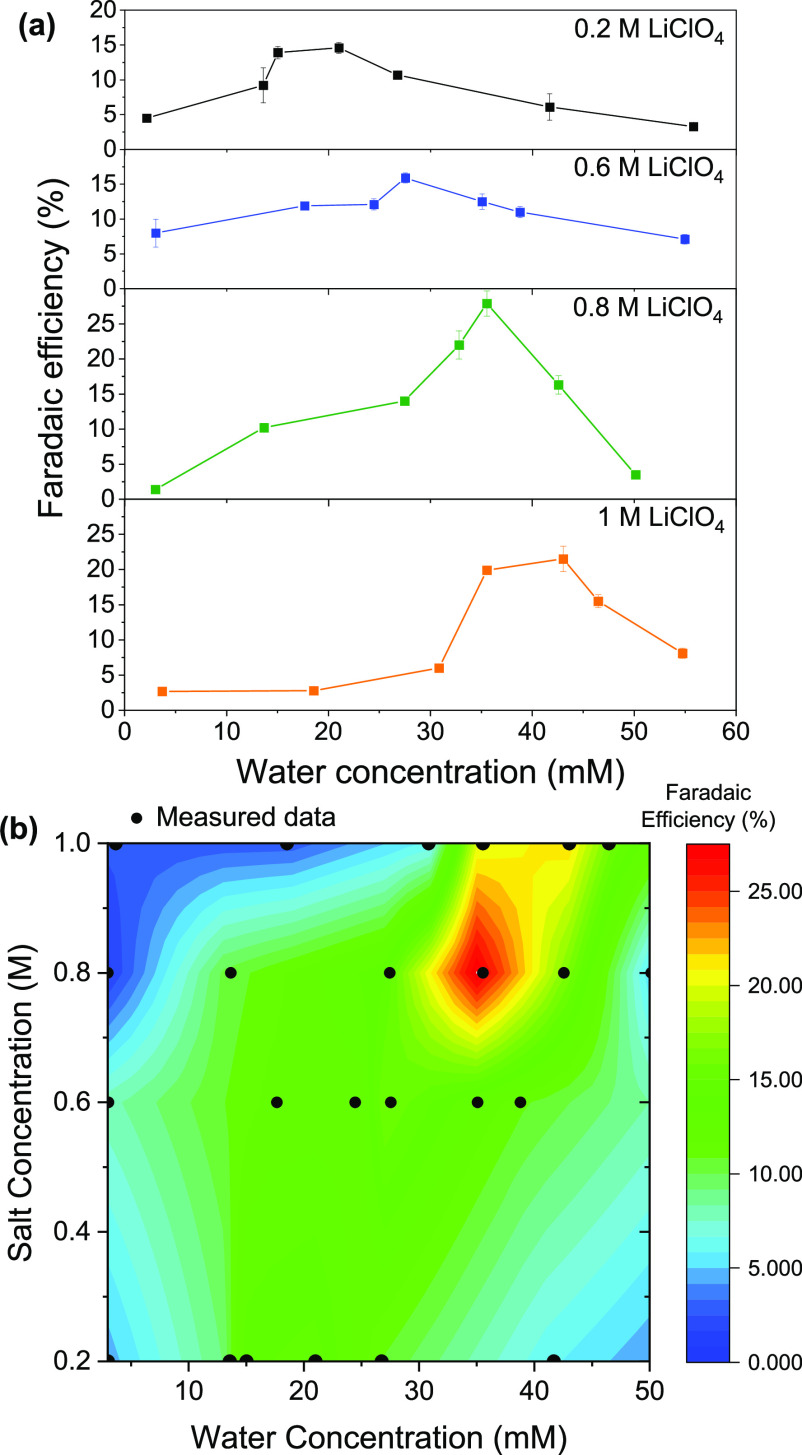
(a) Effect of initial water concentration on Faradaic efficiency in electrolytes of 0.2 M, 0.6 M, 0.8 M, 1 M LiClO_4_ in THF with 1% v/v ethanol. In each experiment, 10 C was passed at a current density of −2 mA cm^–2^. Each data point represents a single experimental measurement. Faradaic efficiency data can be found in Table S1. Error bars represent the standard error calculated from the standard addition method of ammonia quantification (see Supporting Information, Figure S1). (b) A heat map showing the variation in Faradaic efficiency with LiClO_4_ concentration and water concentration, using data from panel a. Intermediate values have been obtained by linear interpolation between measured values. Measured values are shown as black circles.

**Figure 2:**
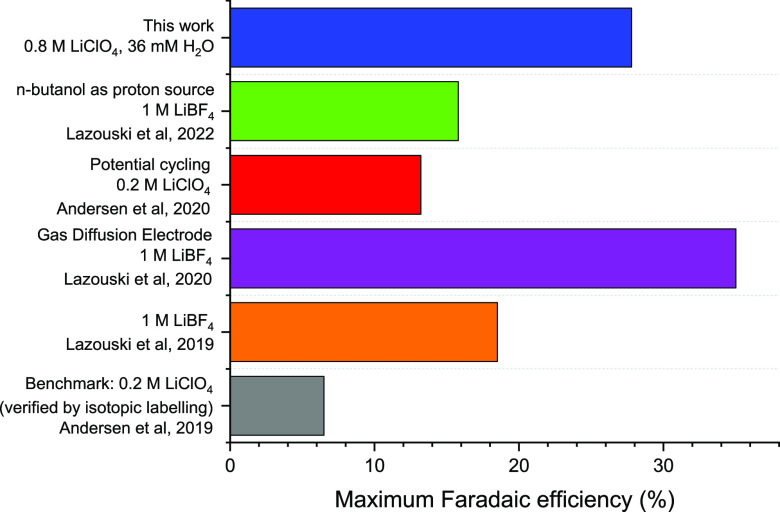
A comparison of the maximum Faradaic efficiencies reported for different strategies at ambient pressure.^2,3,6,18,21^ Each of these systems uses THF as the solvent, and 1% v/v ethanol as a proton source, unless otherwise stated.

We propose that the origin of the improved Faradaic selectivity in the presence of trace H_2_O is the same as that reported by Li et al. for trace O_2_.^20^ They suggested that Li_2_O species formed in the SEI reduced Li^+^ diffusivity in the SEI,^19^ thus kinetically suppressing excessive lithium plating, a significant parasitic side-reaction, as well as other electrolyte-degrading side reactions.^24^ Quantitative electrochemical measurements of suppression of Li plating will be possible given recent developments of more accurate reference electrodes for the Li-mediated system.^25,26^ Our own XPS results are remarkably similar to those of Li et al., as shown in Figure 3a-d: increasing water generally shifts the peaks away from those corresponding to LiClO_n_ under dry conditions (i.e., 533 eV for O 1s and 56.9 eV for Li 1 s) to those corresponding to toward Li_2_O under moist conditions (i.e., 531.5 eV for O 1s and 55.3 eV for Li 1 s. Our results suggest that the replacement of LiClO_n_, LiCl, and related species with nonchlorinated species such as Li_2_O, consistent with earlier reports from the battery literature on the effect of trace H_2_O on the SEI.^4^ Increasing the water concentration beyond the optimum appears to increase surface Li atomic concentration (46% to 54%) and decrease O concentration slightly (41% to 34%), which could indicate increased Li_2_O formation, as shown in Figure 3d. The decrease in Faradaic efficiency at higher water concentrations may be due to excessive proton activity in moist conditions, which favors hydrogen evolution as a side reaction, as measured by Tsuneto et al. at higher ethanol concentrations,^1^ and proposed by Nørskov and co-workers in theoretical work,^15,27^ and may still modify SEI bulk characteristics, such as porosity.^18^ It should be noted that the SEI surface may not be homogeneous, in both composition and morphology;^18,28^ our XPS results likely probe a small section of the SEI’s surface. Our earlier work shows that the composition of the SEI likely varies with depth, particularly for higher salt concentrations.^28^Figure 1b is redolent of the heatmaps of predicted Faradaic efficiency as a function of transport rates proposed by Andersen et al.^6^ We propose that the region of highest efficiency shown in Figure 1b corresponds to the locally optimized relative transport rates.

**Figure 3:**
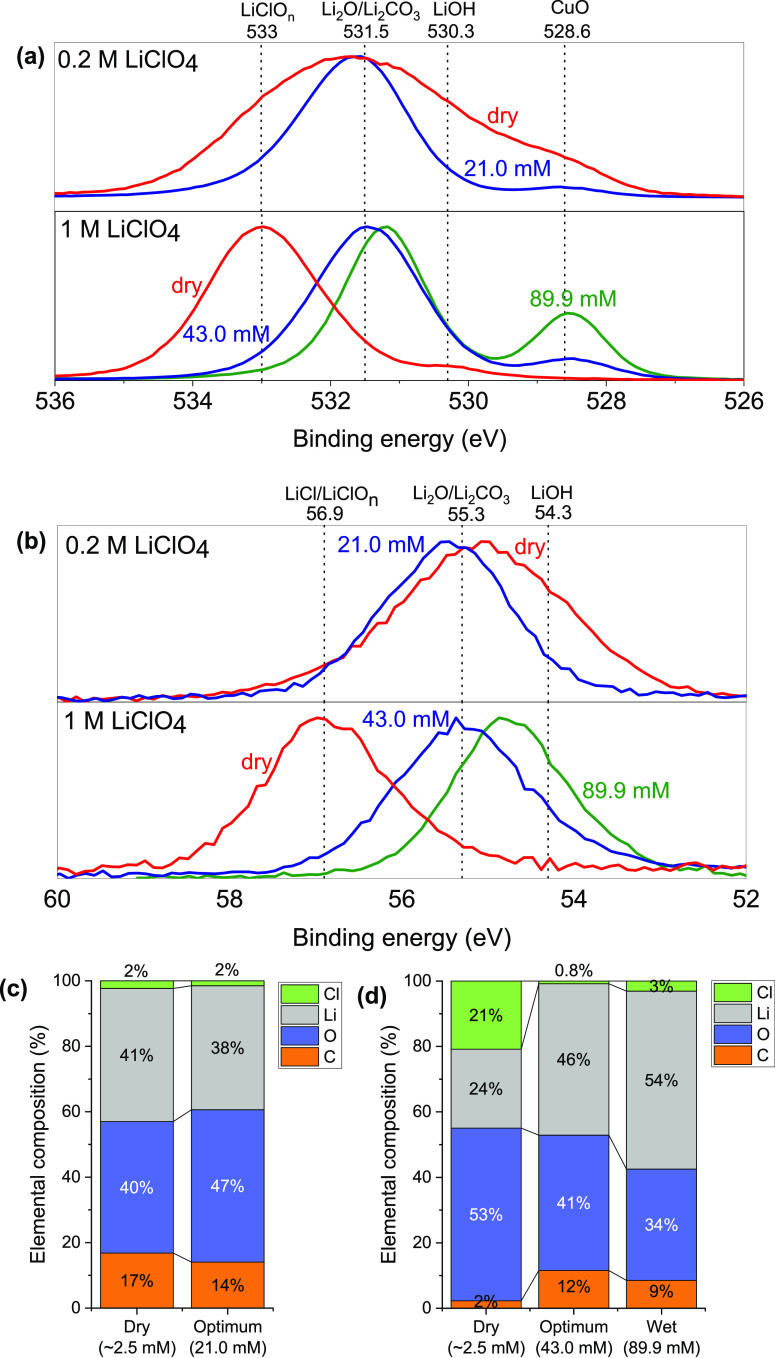
X-ray Photoelectron Spectroscopy measurements: (a) O 1s core level spectra, (b) Li 1s core level spectra, measured for electrode SEIs following experiments in 0.2 and 1 M LiClO_4_ electrolytes, under dry (red line), optimum initial water concentration (blue line) and wetter than optimum (green line) conditions. (c-d) Relative elemental compositions of C, O, Li, and Cl under dry and wet conditions for 0.2 and 1 M LiClO_4_ electrolytes, respectively. Other elements observed in trace amounts have been excluded. H cannot be detected by XPS so is also excluded but will be present in the SEI.^28^ Data for dry samples is replotted from previous work.^28^ All samples were gently rinsed in 0.1 mL of THF after the experiment to remove dried electrolyte from the surface. Spectra are all normalized to their respective peak maxima. Quantitative peak fitting is not presented here due to small differences in binding energy peak positions for Li_2_O and Li_2_CO_3_ and LiClO_n_ species. Attempts at peak fitting to determine specific Li and O species can be found in the Supporting Information (Figures S5, S6).

The peaks in Faradaic efficiency with water concentration are similar in shape to those observed by others upon changing ethanol concentration.^1,3^ The 1% ethanol (0.18 M) used in experiments in our current work—and in most other work in this field^1,3,4,20,21^—is the optimum ethanol concentration reported by Tsuneto et al. in experiments under 50 bar N_2_. Lazouski et al. reported an optimum ethanol concentration of 0.1 M under 1 bar N_2_ using 1 M LiBF_4_ in the electrolyte.^3^ As these reports differ, it is probable that the Faradaic efficiency peak position with ethanol concentration shifts depending on other experimental conditions (e.g., salt type, salt concentration, pressure, current density, etc.). Operating at the tip of such a sharp peak could introduce significant variability in Faradaic efficiency for very small differences in ethanol concentration, i.e., there is ample opportunity for further fine-tuning of the ethanol, salt, and water concentrations. Moreover, the higher selectivity observed with a gas diffusion electrode means that local N_2_ activity at the electrode is suboptimal. We can therefore assume that the use of a gas diffusion electrode as well as optimized electrolyte composition would improve Faradaic efficiency even further.

Until now, prior studies have reported that water contamination led to an unambiguously detrimental effect on selectivity to ammonia. In contrast, we demonstrate that trace water can significantly enhance Faradaic efficiency within a narrow water concentration range of 10–50 mM, with a maximum observed Faradaic efficiency of 27.9 ± 2.5%. This is the highest Faradaic efficiency reported, to the best of our knowledge, at ambient pressure without a gas diffusion electrode. We propose that controlled traces of water in the electrolyte controls the properties of the SEI, by forming compounds with low lithium diffusivity such as Li_2_O, and thus suppressing excessive lithium plating,^20^ while still allowing transport of N_2_ and protons at rates favorable for N_2_ reduction.

We highlight the sensitivity in the lithium-mediated system where different parameters can lead to improvements through minor changes in electrolyte composition. Our controlled fine-tuning of water content reveals that a delicate balance of process parameters is extremely important; the optimum conditions can be easily overlooked. We envisage that even higher Faradaic efficiencies may be possible at ambient pressure in the lithium-mediated system with a more rigorous, systematic approach to optimizing experimental conditions. The research community has discarded many lithium free systems on the basis that they are inactive,^1,2^ under very specific experimental conditions. From our current results, which show how highly sensitive the Li-mediated system is to water, we conjecture that the experimental parameters of the other systems may have been so far from optimum that they appear inactive, and could be worth revisiting; for example, these thus far inactive systems could be enhanced through wider screening of electrolyte parameters, incorporating an artificial SEI or adding other interfacial modifications to slow down proton transport and inhibit hydrogen evolution. Electrolytes and interphases that would tailor access to protons and N_2_—yet not constrained by the lithium plating potential—would lead to step changes in the energy efficiency of electrochemical N_2_ reduction.

## Supporting Information Available

Materials, ammonia quantification, water concentration measurements, working electrode stability, experiments list, XPS measurements (PDF)
