# Supplementary material for: Water Increases the Faradaic Selectivity of Li-Mediated Nitrogen Reduction
Source: ACS Energy Lett. 2023 Jan 31;8(2):1230–5. doi: 10.1021/acsenergylett.2c02792 (PMC9926485; doi:10.1021/acsenergylett.2c02792)
Supplement: Supplementary file 1 — nz2c02792_si_001.pdf [file nz2c02792_si_001.pdf]

Water increases the Faradaic selectivity of Li-mediated nitrogen reduction – Supporting Information

Matthew Spry[1], Olivia Westhead[1], Romain Tort[2], Benjamin Moss[1], Yu Katayama[3], Maria-Magdalena Titirici[2], Ifan E. L. Stephens[1], Alexander Bagger[2]

[1] Department of Materials, Imperial College London, Prince Consort Road, South Kensington, London, SW7 2AZ, UK

[2] Department of Chemical Engineering, Imperial College London, Imperial College Rd, South Kensington, London, SW7 2AZ, UK

[3] SANKEN (Institute of Scientific and Industrial Research), Osaka University, Mihogaoka, Ibaraki, Osaka 567-0047, Japan

1. Materials

In each experiment, the working electrode was 1 cm^2 Mo foil (Goodfellow, 99.9%, 0.125 mm thick) with Cu wire as a current collector (Goodfellow, 99.99% 0.5 mm diameter), the counter electrode was Pt mesh (Goodfellow, 99.9%) with approximately 1 cm^2 geometric area, and the counter current collector and reference electrode were Pt wire (Goodfellow, 99.99%, 0.5 mm thick). Before the cell is assembled, the Mo working electrode was dipped in 4 M HCl, rinsed in ethanol and then polished with 400, 1500 and 2500 grit silicon carbide paper to a mirror finish, and then sonicated for 10 minutes in ethanol. The counter and reference electrodes were flame annealed. All experiments and electrolyte preparations were performed inside an Ar-filled MBraun glove box. Experiments were conducted with water concentrations between 3 and 55 mM, at different $LiClO_4$ (Alfa-Aesar, 99%, anhydrous) concentrations of 0.2 M, 0.6 M, 0.8 M and 1 M in 99% tetrahydrofuran (Sigma Aldrich, anhydrous, >99.9%, inhibitor-free) and 1% ethanol (Sigma Aldrich, 99.9%, Extra Dry, AcroSeal) by volume. The water concentration was varied by adding 1600 ppm ultra-pure water (>18.2 MΩ, Sartorius) to one THF bottle and using appropriate mixtures of "dry" and "wet" THF. A sample of electrolyte was then removed and stored in a septum vial to measure the water concentration. All experiments were conducted in a custom-made glass three-electrode electrochemical cell containing 12.5 ml of electrolyte. Prior to each experiment, Ar (BOC, N6) was bubbled though the electrolyte at 20 ml/min to test for leaks, then N_2 (BOC, N6) was bubbled through at 20 ml/min for 30 mins to pre-saturate the electrolyte. Purifiers for Ar and N_2 were purchased from NuPure to remove H_2O, H2, CO2, O2, CO, nonmethane hydrocarbon (NMHC), CH4, NH3, NOx to levels of less than 0.5 ppb.

After presaturation with N_2, the gas flow rate was reduced to 5 ml/min for the duration of the experiment. For each electrochemistry experiment, the open circuit potential (OCP) first was measured for 30 seconds, then potentiostatic impedance spectroscopy (PEIS) was performed to measure the electrolyte resistance, and a linear sweep voltammogram (LSV) was measured from 0 V to until the onset of lithium plating (normally -3.6 V for 0.8 M and 1 M LiClO4 electrolytes, and -4 V for 0.2 M and 0.6 M electrolytes). Chronopotentiometry (CP) was run at a current density of -2 mA/cm^2 until 10 C of charge was passed, followed by a second PEIS and OCP measurement.

After the experiment, Ar was bubbled through the cell at 20 ml/min to purge the cell of nitrogen before dissembling the cell. A second sample of electrolyte was taken and stored in a septum vial to later measure the water concentration. Approximately 4 ml of electrolyte was transferred to another vial for ammonia quantification. The cell and electrolyte samples were then taken out of the glove box. The cell body, platinum electrodes, magnetic stirrer and rubber stoppers were then boiled for 1 hour before being dried in an oven at 70 degrees overnight.

2. Ammonia quantification

Ammonia quantification was performed using the indophenol blue method with standard addition of a known quantity of ammonium chloride solution, similar to that described by Cherepanov et al[1].

Stock 0.4 M solution was made using 800 mg of NaOH pellets (VWR) in 50 ml ultra-pure water, 12% Sodium hypochlorite solution and phenol nitroprusside solution (Sigma Aldrich) were made/purchased and stored at 4°C.

For each experiment, 8 equal samples of electrolyte (400 µl for 0.2 M and 0.6 M electrolytes, and 200 µl for 0.8 M and 1 M) were taken and 4 M HCl was added (20 µl to 400 µl samples and 10 µl to 200 µl samples) to convert any NH_3 to NH_4Cl. One sample of blank electrolyte was also prepared in this way. These samples were then heated in a water bath at approximately 68°C until the solvent had evaporated. The remaining residue was then diluted in 2 ml of ultra-pure water. Smaller volumes of electrolyte for 0.8 M and 1 M salt concentrations were used as the standard addition method of quantification relies on linearity between ammonia concentration and absorbance. We observed, in agreement with Giner-Sanz et al[2], that $LiClO_4$ interferes with the indophenol process. The calibration curves became non-linear for electrolytes of 0.8 M $LiClO_4$ and above using this method. Using a smaller electrolyte volume kept the final indophenol solution concentration low enough to ensure linearity. The resulting aqueous solutions were transferred to 3 ml cuvettes.

Standard addition samples were made by adding 10, 15, 20 and 25 µl of a 500 ppm aqueous NH_4Cl solution to four of the cuvettes before adding the indophenol reagents. The sodium hypochlorite and sodium hydroxide solutions were mixed in a 9:1 ratio immediately before a measurement to create the required alkaline solution. 500 µl of each of phenol nitroprusside solution and alkaline solution were added to all but one cuvette, to which 1 ml ultra-pure water was added to make a background sample. The cuvettes were covered with aluminium foil and left in the dark for 30 mins.

The UV absorbance of the resulting samples was measured in a UV-vis spectrometer between 400 and 900 nm with background correction from the background sample. The absorbance value was taken as the difference between the peak absorbance (observed around 635 nm) and the absorbance and 900 nm. The absorbance of the blank electrolyte was subtracted from the absorbances of the other samples.

The sample absorbance was plotted against "added" ammonia (in moles, from adding NH_4Cl solution), with the regular electrolyte samples lying on the y-axis and forming a straight line through the standard addition points. The x-intercept of the line gives the moles of ammonia in the regular cuvette. From this value, the total number of moles in the electrolyte was calculated. The error in the number of moles in the electrolyte was derived from the standard error of the x-intercept.

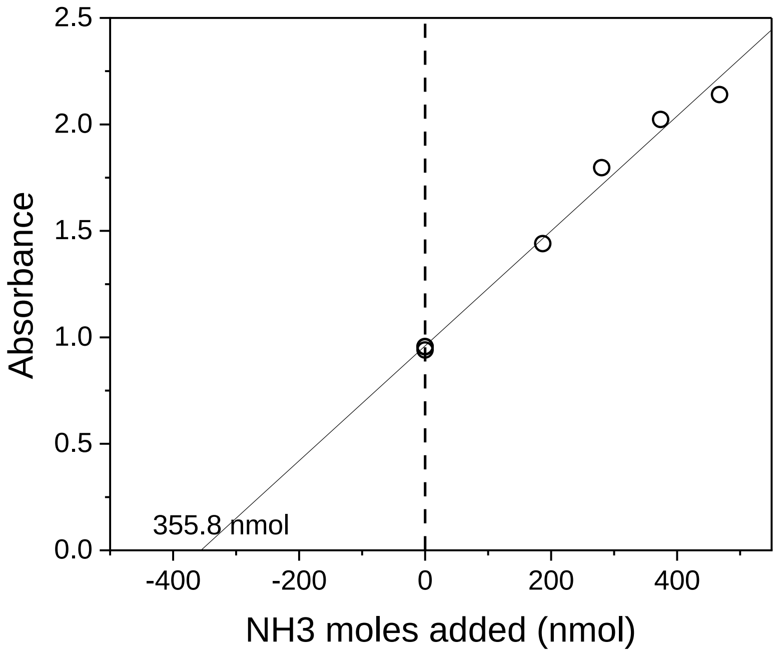

Figure S1: Standard addition method of quantification of ammonia in a sample of electrolyte. Three electrolyte sample measurements are plotted with zero added ammonia, and four measurements with known amounts of added ammonia. Extrapolating the line through these points back to the x-axis gives the number of moles of ammonia present in each sample.

The Faradaic efficiency of each experiment was calculated using the following equation:

$$FE_{NH_3}(\%) = \frac{3\,F\,n}{Q}$$

where F is the Faraday constant, n is the number of moles in the electrolyte, and Q is the total charge passed during the experiment.

Blank experiments passing both Ar and N_2 were performed to test for contamination, as per the protocol described by Andersen et al[3]. The ability of this system to produce ammonia is well documented, so isotopically labelled experiments were not performed. For the Ar blanks, the experiments procedure was identical to that of a nitrogen reduction experiment, except that Ar was passed instead of N2. For N2 blanks, the N2 was passed at open-circuit potential for the same time period as a regular N2 reduction experiment. Negligible ammonia was detected in any blank experiment, as shown in Table 1.

3. Water concentration measurements

For each experiment, a 2.5 ml sample of electrolyte was taken before and after electrochemical measurements. These were initially stored in septum vials in the glove box, then later removed and tested in a Karl-Fisher titrator. The titrator reading is ppm, given as µg water per g solution, which is converted to mM in Figure S2, to account for differences in solution density at different salt concentrations. Generally, an increase of around 100 ppm was observed over the course of these experiments, though samples which were left for longer periods before being tested yielded greater increases in measured water concentration, which may suggest a homogeneous water-forming reaction takes place in the electrolyte after the experiment.

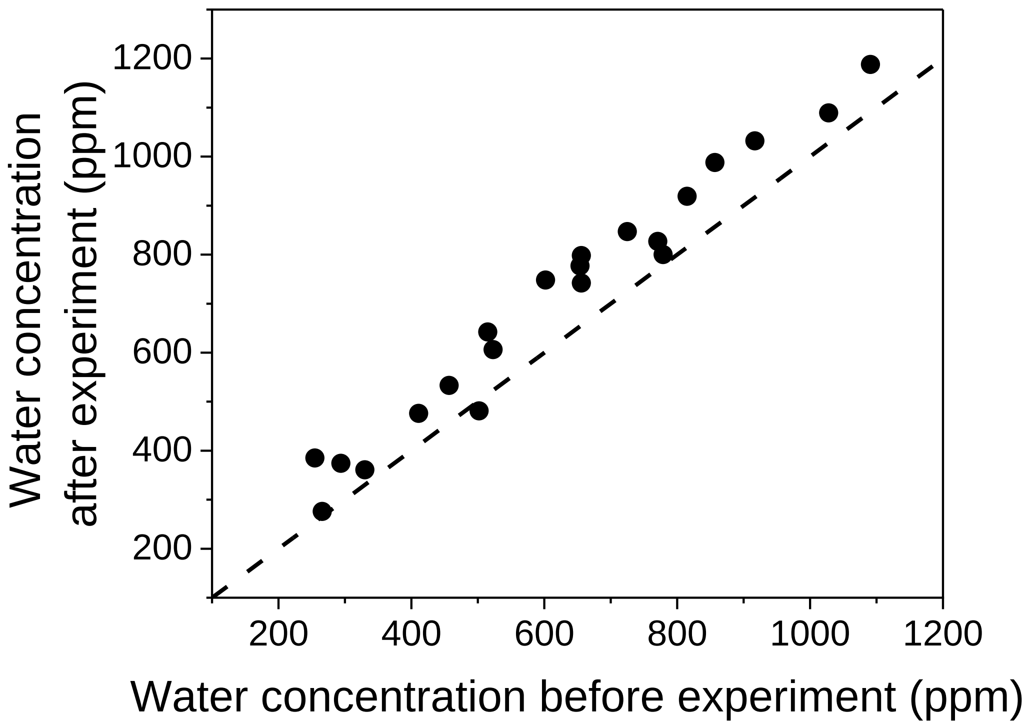

Figure S2: Water concentration at the start of an experiment vs water concentration at the end. The dashed black line (y = x) represents no change in water concentration.

4. Working electrode stability

As reported in out earlier work[4], the working electrode potential instability observed in $LiClO_4$ electrolytes by Andersen et al[5] did not occur when the salt concentration was above 0.6 M, which was attributed to a more stable, inorganic, salt-based SEI forming, instead of a largely organic, solvent-based SEI. In these experiments, significant drift of the working electrode towards more negative potentials during chronopotentiometry was only observed in a 0.2 M dry electrolyte. Stable electrode potentials were observed throughout experiments with 0.2 M $LiClO_4$ electrolytes for all experiments with added water above 266 ppm. Figure S3 shows the possible onset of working electrode instability for 266 ppm water in a 0.2 M $LiClO_4$ electrolyte near the end of the experiment, but the potential is stable for around one hour.

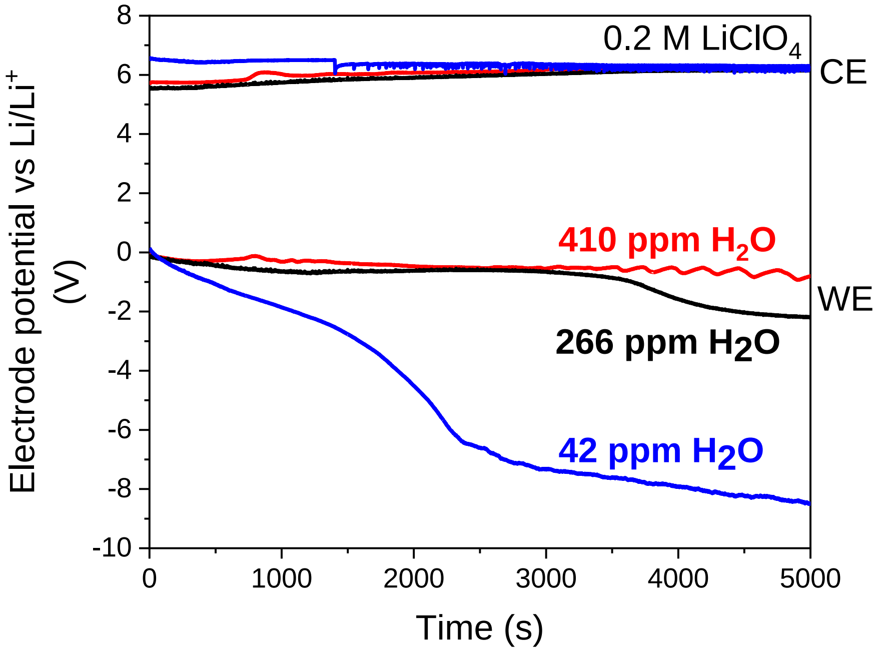

Figure S3: Chronopotentiometry of nitrogen reduction experiments in 0.2 M LiClO4 electrolytes with different water concentrations (42 ppm, 266 ppm, 410 ppm). All experiments passed 10 C of charge with a current of -2 mA.

5. Experiments

Table S1: List of experiments varying water and salt concentration, showing water concentration before and after the experiment, and the Faradaic efficiency. The error value shown is derived from the standard error obtained from the linear regression model used in the standard addition method described in section 2.

Expt No.	$LiClO_4$ conc.	H_2O conc. (before)		H_2O conc. (after)		FE	Error
	M	ppm	mM	ppm	mM	%	%
1	0.6	1028	54.7	1089	58.2	7.1	0.7
2	0.6	725	38.8	847	45.3	11	0.8
3	0.6	515	27.5	642	34.4	15.9	0.7
4	0.6	330	17.7	361	19.3	11.9	1.2
5	0.6	57	3.0			8.0	2.0
6	0.2	1091	55.8	1188	60.7	3.3	0.1
7	0.2	815	41.7	919	47.0	6.1	1.9
8	0.2	523	26.8	606	31.0	10.7	0.3
9	0.2	294	15.0	374	19.1	13.9	0.9
10	0.2	42	2.1			4.5	0.4
11	1	980	54.7			8.1	0.7
12	1	771	43.0	827	46.2	21.5	1.8
13	1	552	30.8			6.0	0.3
14	1	332	18.5			2.8	0.1
15	1	66	3.7			2.7	0.2
16	0.6	457	24.5	533	28.5	12.1	0.8
17	0.2	410	21.0	476	24.3	14.6	0.8
18	0.2	266	13.6	276	14.1	9.2	2.5
19	0.6	656	35.1	798	42.7	12.5	1.1
21	0.8	917	50.1	1032	56.4	3.5	0.4
22	0.8	779	42.6	800	43.7	16.3	1.3
23	0.8	502	27.5	481	26.3	14.0	0.5
24	0.8	255	13.9	385	21.0	10.2	0.6
25	0.8	53	2.9			1.4	0.1
26	1	654	35.8	777	43.4	19.9	0.4
27	1	857	46.9	988	55.2	15.5	0.9
28	0.8	656	35.9	742	40.6	27.9	2.5
29	0.8	602	32.9	748	40.9	22.0	1.9
B1-Ar	0.2	500*	27.3*			0.1	0.0
B2-N_2	0.2	500*	27.3*			0.1	0.0
B3-Ar	0.8	500*	27.3*			0.3	0.1
B4-N_2	0.8	500*	27.3*			0.1	0.0

6. Water concentration reports in literature

The effect of water concentration on the lithium-mediated system has been mentioned in several reports in literature, but not previously systematically studied. In the seminal paper on this system, Tsuneto et al reported that water was not a feasible proton source and measured 10 mM (approx. 200 ppm) water in their electrolytes using ethanol as a proton source[6]. Lazouski et al studied the effect of adding in excess of 60 mM water, showing that this significantly reduced Faradaic efficiency to ammonia[7]. However, these experiments were not conducted inside a glove box, and the water content of the purportedly dry electrolyte was not reported. Their as-bought THF was reported to contain 30 mM (approx. 600 ppm) water, so if this was not dried, their electrolytes would have had water concentrations within the range tested in this report. Cherepanov et al observed variations in the water content of their electrolytes between 0.3 mM and 3 mM, proposing that this could have been a source of variability in their results[1]. Krishnamurthy et al reported no significant correlation between the water content of a range of different proton donors, and the maximum Faradaic efficiency observed using that proton donor, though they did not the total water content of the electrolyte vs Faradaic efficiency[8].

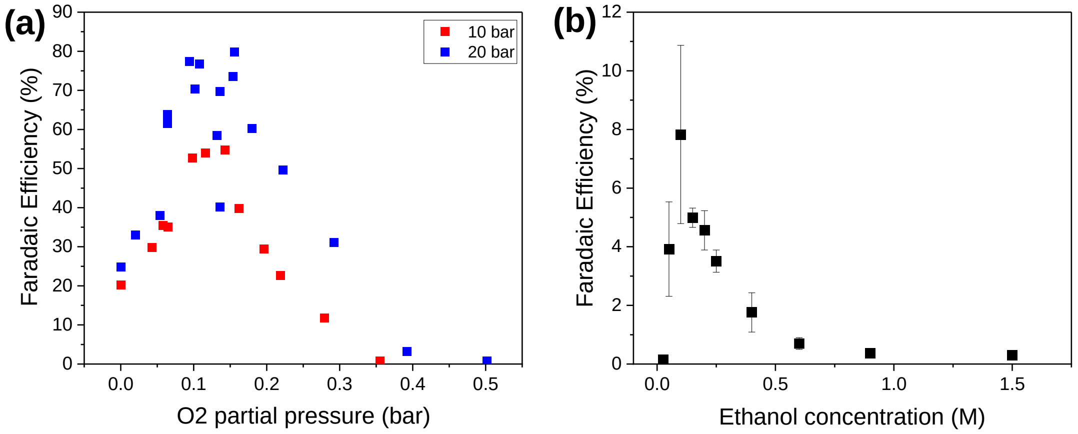

Figure S4: (a) Faradaic efficiency as a function of O2 partial pressure (replotted from Li et al[9]), (b) Faradaic efficiency as a function of ethanol concentration (replotted from Lazouski et al[7]).

Trends in Faradaic efficiency with water concentration show remarkable similarities to those of other parameters, such as O2 concentration and ethanol concentration, as shown in Figure S4. It is possible that some or all of these factors are all related to the same phenomenon, for example the improvement in Faradaic efficiency with O_2 could be related to the formation of water at the cathode. Similarly, the water in these experiments could be oxidised to O_2 at the anode. Nonetheless, the sharpness of these peaks highlights the importance of rigorous and systematic studies of the effects of multiple parameters. Careful optimisation of multiple parameters could allow efficient nitrogen reduction at ambient pressure.

Using O_2 in THF solubility data available in literature[10] (saturation mole fraction of 8×10^{-4} under 1 bar O_2) the approximate O_2 concentration in the electrolyte at peak Faradaic efficiency (under approx. 0.1 bar O_2 partial pressure) in the report from Li et al[9] is around 1 mM. Assuming all the O_2 in a saturated electrolyte is reduced to water, this corresponds to a total electrolyte concentration of 2 mM. Li et al report initial water concentrations of 30-40 ppm (approx. 1.5-2 mM) and increases of 30-70 ppm (approx. 1.5-3.5 mM) water over most experiments, passing 50 C of charge. The optimum water concentration at peak Faradaic efficiency in O_2 experiments does not align with the optimum water concentration observed in our experiments. It should be noted however that our experiments were

performed under 1 bar N_2, whereas those of Li et al were performed under 10 or 20 bar N_2. If, as we believe, the role of water is to modify the SEI to optimise transport rates of N_2, Li^+ and protons, then at higher N2 pressure, the conditions for optimum transport may also be vastly different.

7. XPS

0.2 M LiClO4

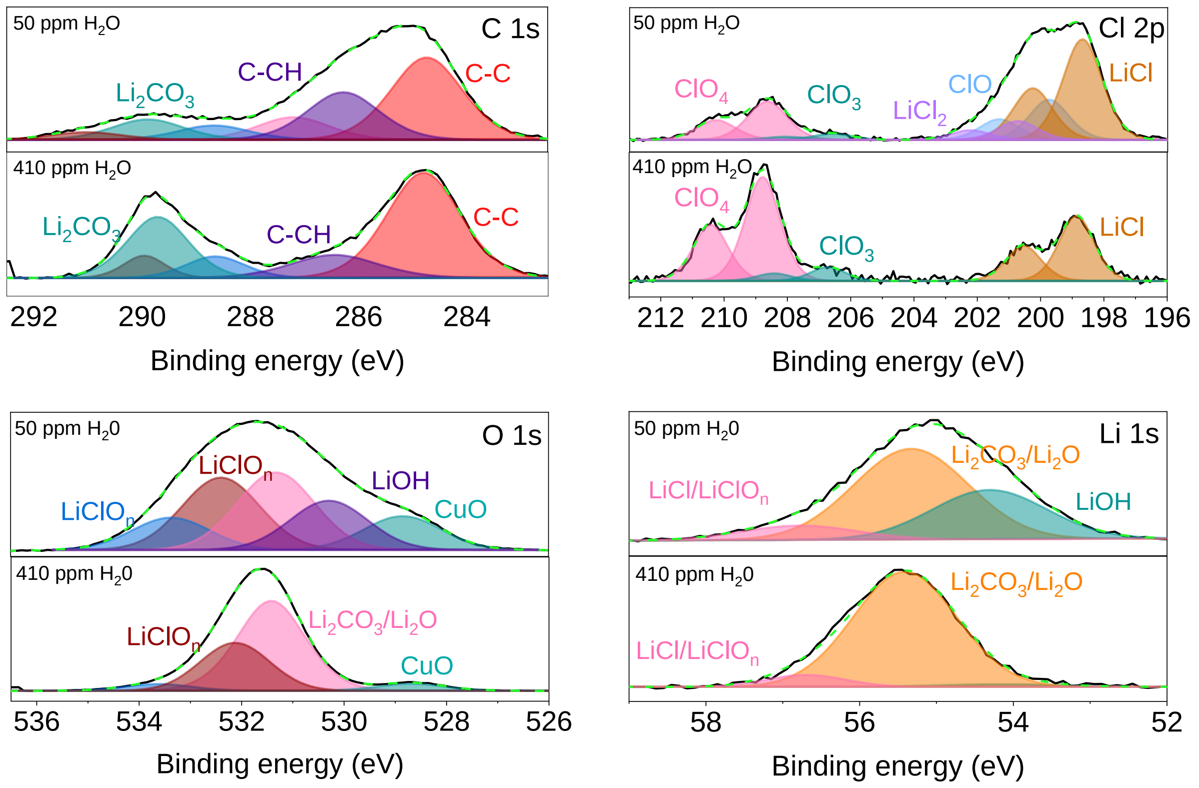

Figure S5: XPS spectra of the working electrode SEI from a 0.2 M $LiClO_4$ electrolyte with 50 ppm water (dry) and 410 ppm water (optimum). Attempted peak fittings to are shown by the shaded peaks. The measured XPS spectrum is shown as a black line, and the envelope of fitted peaks is shown as a green dashed line.

1 M $LiClO_4$

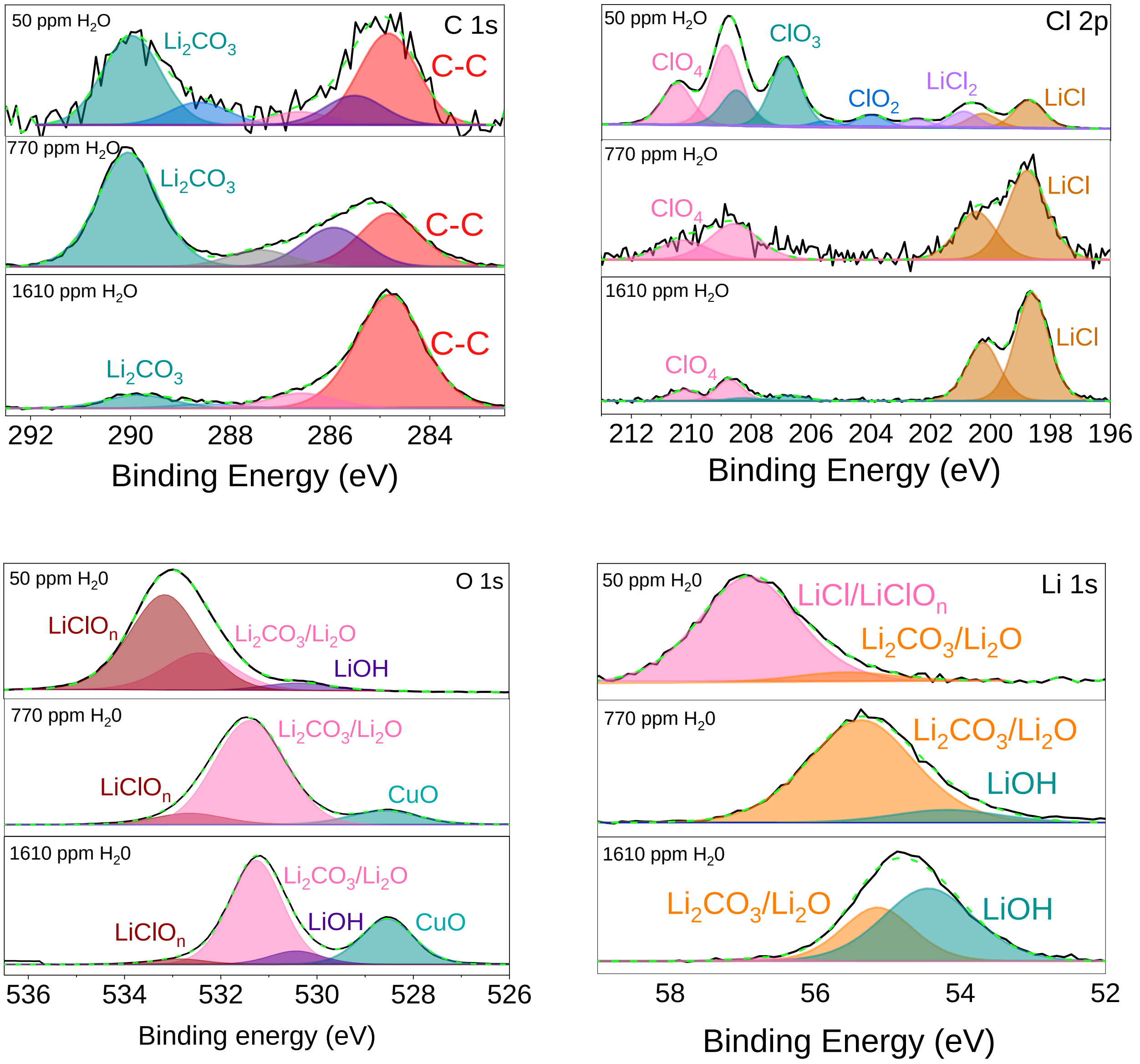

Figure S6: XPS spectra of the working electrode SEI from a 1 M $LiClO_4$ electrolyte with 50 ppm water (dry) and 770 ppm water (optimum) and 1610 ppm (higher than optimum). Attempted peak fittings to are shown by the shaded peaks. The measured XPS spectrum is shown as a black line, and the envelope of fitted peaks is shown as a green dashed line.

XPS measurements (Figure S5, S6) were taken on electrodes from experiments with 0.2 M and 1 M $LiClO_4$ at their optimum water concentrations, and one experiment with 1 M salt and a higher than optimum 80 mM (1610 ppm) water concentration. These have been compared to similar measurements on electrodes from dry electrolytes reported in previous work[4]. The O 1s and Li 1s spectra in Figure 3a shows clear differences in surface SEI composition between wet and dry conditions. Figures S5 and S6 show narrowing of both the O 1s and Li 1s peaks for 0.2 M experiments, which imply a smaller range of oxygenated and lithiated species focussing on Li_2O and Li_2CO_3 (more likely Li_2O), and a clear shift towards this peak position for 1 M experiments. In 1 M $LiClO_4$ experiments, the approximately 20%

chlorine content observed in dry conditions[4] (see Figure 3d) which suggests a heavily LiCl and $LiClO_n$-based SEI, is not observed in experiments with higher water content, though it is possible that some of the $LiClO_4$ observed may have arisen from dried electrolyte still present on the surface. For the SEIs made in wet electrolytes, the oxygen content by atomic percentage also decreases, and lithium content approximately doubles. The O 1s and Li 1s XPS spectra suggest a strong shift from $LiClO_n$ under dry conditions towards Li_2O. This could be due to the replacement of $LiClO_n$, LiCl and related species with non-chlorinated species such as Li_2O, which has much higher relative lithium content. Increasing the water concentration above the optimum appears to make only small further modifications to the SEI elemental composition, though may still modify the SEI bulk characteristics, such as porosity[11].

8. References

1. Cherepanov P v., Krebsz M, Hodgetts RY, Simonov AN, Macfarlane DR. Understanding the Factors Determining the Faradaic Efficiency and Rate of the Lithium Redox-Mediated N2Reduction to Ammonia. *Journal of Physical Chemistry C*. 2021;125(21):11402-11410. doi:10.1021/acs.jpcc.1c02494

2. Giner-Sanz JJ, Leverick GM, Giordano L, Pérez-Herranz V, Shao-Horn Y. Alkali Metal Salt Interference on the Salicylate Method for Quantifying Ammonia from Nitrogen Reduction. *ECS Advances*. 2022;1(2):024501. doi:10.1149/2754-2734/ac6a68

3. Andersen SZ, Čolić V, Yang S, et al. A rigorous electrochemical ammonia synthesis protocol with quantitative isotope measurements. *Nature*. 2019;570(7762):504-508. doi:10.1038/s41586-019-1260-x

4. Westhead O, Spry M, Bagger A, et al. The Role of Ion Solvation in Lithium Mediated Nitrogen Reduction. *J Mater Chem A,* 2022. doi:10.1039/D2TA07686A

5. Andersen SZ, Statt MJ, Bukas VJ, et al. Increasing stability, efficiency, and fundamental understanding of lithium-mediated electrochemical nitrogen reduction. *Energy Environ Sci*. 2020;13(11):4291-4300. doi:10.1039/d0ee02246b

6. Tsuneto A, Kudo A, Sakata T. Lithium-mediated electrochemical reduction of high pressure N2 to NH 3. *Journal of Electroanalytical Chemistry*. 1994;367:183-188.

7. Lazouski N, Schiffer ZJ, Williams K, Manthiram K. Understanding Continuous Lithium-Mediated Electrochemical Nitrogen Reduction. *Joule*. 2019;3(4):1127-1139. doi:10.1016/j.joule.2019.02.003

8. Krishnamurthy D, Lazouski N, Gala ML, Manthiram K, Viswanathan V. Closed-Loop Electrolyte Design for Lithium-Mediated Ammonia Synthesis. *ACS Cent Sci*. 2021;7(12):2073-2082. doi:10.1021/acscentsci.1c01151

9. Li K, Andersen SZ, Statt MJ, et al. Enhancement of lithium-mediated ammonia synthesis by addition of oxygen. *Science*. 2021;374:1593-1597.

10. Battino Rubin. *Solubility Data Series: Oxygen and Ozone*. Pergamon; 1981.

11. Lazouski N, Steinberg KJ, Gala ML, Krishnamurthy D, Viswanathan V, Manthiram K. Proton Donors Induce a Differential Transport Effect for Selectivity toward Ammonia in Lithium-Mediated Nitrogen Reduction. *ACS Catal*. 2022;12(9):5197-5208. doi:10.1021/acscatal.2c00389
